# Atp6i deficient mouse model uncovers transforming growth factor-β1 /Smad2/3 as a key signaling pathway regulating odontoblast differentiation and tooth root formation

**DOI:** 10.1038/s41368-023-00235-2

**Published:** 2023-08-21

**Authors:** Jue Wang, Abigail McVicar, Yilin Chen, Hong-Wen Deng, Zhihe Zhao, Wei Chen, Yi-Ping Li

**Affiliations:** 1 Department of Pathology, School of Medicine, University of Alabama at Birmingham, Birmingham, AL USA; 2 State Key Laboratory of Oral Diseases & National Center for Stomatology & National Clinical Research Center for Oral Diseases & Department of Orthodontics, West China Hospital of Stomatology, Sichuan University, Chengdu, China; 3 Division in Cellular and Molecular Medicine, Department of Pathology and Laboratory Medicine, Tulane University School of Medicine, Tulane University, New Orleans, LA USA; 4 Tulane Center of Biomedical Informatics and Genomics, Deming Department of Medicine, Tulane University School of Medicine, New Orleans, LA USA

**Keywords:** Differentiation, Growth factor signalling

## Abstract

The biomolecular mechanisms that regulate tooth root development and odontoblast differentiation are poorly understood. We found that *Atp6i* deficient mice (*Atp6i*^*−/−*^) arrested tooth root formation, indicated by truncated Hertwig’s epithelial root sheath (HERS) progression. Furthermore, Atp6i deficiency significantly reduced the proliferation and differentiation of radicular odontogenic cells responsible for root formation*. Atp6i*^*−/−*^ mice had largely decreased expression of odontoblast differentiation marker gene expression profiles (*Col1a1, Nfic, Dspp, and Osx*) in the alveolar bone. *Atp6i*^*−/−*^ mice sample RNA-seq analysis results showed decreased expression levels of odontoblast markers. Additionally, there was a significant reduction in Smad2/3 activation, inhibiting transforming growth factor-β (TGF-β) signaling in *Atp6i*^*−/−*^ odontoblasts. Through treating pulp precursor cells with *Atp6i*^*−/−*^ or wild-type OC bone resorption-conditioned medium, we found the latter medium to promote odontoblast differentiation, as shown by increased odontoblast differentiation marker genes expression (*Nfic, Dspp, Osx*, and *Runx2)*. This increased expression was significantly blocked by anti-TGF-β1 antibody neutralization, whereas odontoblast differentiation and Smad2/3 activation were significantly attenuated by *Atp6i*^*−/−*^ OC conditioned medium. Importantly, ectopic TGF-β1 partially rescued root development and root dentin deposition of *Atp6i*^*−/−*^ mice tooth germs were transplanted under mouse kidney capsules. Collectively, our novel data shows that the prevention of TGF-β1 release from the alveolar bone matrix due to OC dysfunction may lead to osteopetrosis-associated root formation via impaired radicular odontoblast differentiation. As such, this study uncovers TGF-β1 /Smad2/3 as a key signaling pathway regulating odontoblast differentiation and tooth root formation and may contribute to future therapeutic approaches to tooth root regeneration.

## Introduction

Despite playing an important role in normal physiological function, there is a limited understanding of the cellular, molecular, and genetic regulatory processes that regulate tooth root development. As aberrations in oral health can play profound effects on systemic health, it is crucial to investigate the mechanism(s) underlying how osteoclasts (OCs) participate in root development regulation and how OC dysfunction leads to disrupted tooth root formation. Atp6i [ATPase, H^+^ transporting, (vacuolar proton pump) member I] is one component of the proton pump used by OCs to acidify the surface of and subsequently degrade bone.^1,2^ We previously cloned Atp6i, which encodes the putative osteoclast-specific proton pump subunit, termed OC-116kD.^3^ Atp6i is specifically expressed in differentiated OCs and is critical for OC activity. Human TCIRG1 mutations, encoding *ATP6i*, are associated with autosomal recessive osteopetrosis type I (OPTB1, OMIM #259700).^1,4–6^ We previously generated a mouse model of human osteopetrosis through *Atp6i* deletion (*Atp6i*^*−/−*^ mice),^7^ providing a useful tool for elucidating the mechanism behind root formation defects. Furthermore, our more recent studies have demonstrated that *Atp6i* can be targeted to prevent both bone loss and inflammation in mouse models of periodontal disease^8^ and periapical disease,^9^ implicating *Atp6i* as a key target in oral disease through its important role in osteoclast activity. Additionally, our previous publications have demonstrated the efficacy of targeting other osteoclast proton pump subunits including Atp6v0d2,^10^ Atp6v1c1,^11^ and ATP6AP1 (Ac45).^12,13^

Tooth root development is initiated at post-natal day 4 (PN4) and continues to lengthen for approximately 3 weeks.^14^ Root development requires the cytodifferentiation and maintenance of radicular (root) odontoblasts, which express various transcription factors and dentin matrix markers, including dentin sialophosphoprotein (Dspp), nuclear factor I C (Nfic), osterix (Osx), and nestin, all of which are critical in the process of cytodifferentiation and extracellular matrix formation. Moreover, root formation is initiated through interactions between the dental epithelial bilayer, known as Hertwig’s epithelial root sheath (HERS), and neural crest-derived mesenchyme tissues, the internal dental papilla and external dental follicle.^15^ Various signaling pathways that function during crown formation, including bone morphogenetic protein (BMP), transforming growth factor-β (TGF-β), fibroblast growth factors (FGFs), sonic hedgehog (Shh), and Wnt, are also involved in regulating tooth root development.^16–20^ TGF-β signaling is believed to play essential roles in root development and the formation of the dentin extracellular matrix (DECM) through regulating dental epithelium and mesenchyme interactions.^19,21,22^ Within the TGF-β signaling pathway, receptor-regulated Smad proteins (R-Smads), specifically Smad2 and Smad3, are first activated by a phosphorylation signal that is initiated by the interaction of TGF-β with TGF-β type I and type II receptors (Tgfbr1 and Tgfbr2). After interaction with common Smad (Co-Smad) and Smad4, R-Smads transverse the nuclear membrane before regulating the transcription of target genes via the recruitment of necessary co-factors.^23^ Deletion of the TGF-β signaling family members, such as *Tgfbr2 and Smad4*, results in abnormal radicular dentin formation and molar root shape.^24,25^

Recently, the communication or crosstalk between osteoblasts and OCs has been extensively investigated.^26,27^ Growth factors that are released during osteoclastic bone resorption play a key role in mediating this osteoblast-OC relationship, with TGF-β1 acting as a primary coupling growth factor that when released from the bone matrix promotes bone formation.^28^ As skeletal and dental development share similar biochemical and physiological properties, osteoblasts and odontoblasts exhibit similar functionality within bone and dentin respectively, with the expression of common proteins including bone sialoprotein, type I collagen, and osteocalcin.^29^ Thus, the potential regulatory roles of OCs on odontoblast differentiation may reveal the mechanisms underlying how root formation and odontoblast differentiation are regulated.

Here, are the first to reveal the novel function of TGF-β1 is a key regulator of odontoblast differentiation and tooth root formation. We utilized Atp6i^−/−^ mice as an osteopetrosis disease model to study tooth root formation and found that Atp6i^−/−^ mice displayed impaired tooth root formation with truncated HERS progression. Smad2/3 activation was significantly attenuated indicating suppressed transforming growth factor-β (TGF-β) signaling in Atp6i-deficient odontoblasts. Conditioned medium containing TGF-β1 promoted odontoblast differentiation, while it was significantly blocked by anti-TGF-β1 antibody neutralization, whereas odontoblast differentiation and Smad2/3 activation were significantly attenuated by *Atp6i-*deficient OC conditioned medium. Importantly, ectopic TGF-β1 partially rescued root development and root dentin deposition of *Atp6i*^*−/−*^ teeth with transplanted germs from the mouse kidney capsule. Collectively, our data indicates that TGF-β1 is a key regulator of odontoblast differentiation and tooth root formation. This study provides important insights into the mechanisms underlying radicular odontoblast differentiation and root formation and may contribute to novel therapeutic approaches to tooth root regeneration.

## Results

### *Atp6i*^*-/-*^ mice exhibit disrupted tooth root formation with truncated HERS progression

**Fig. 1:**
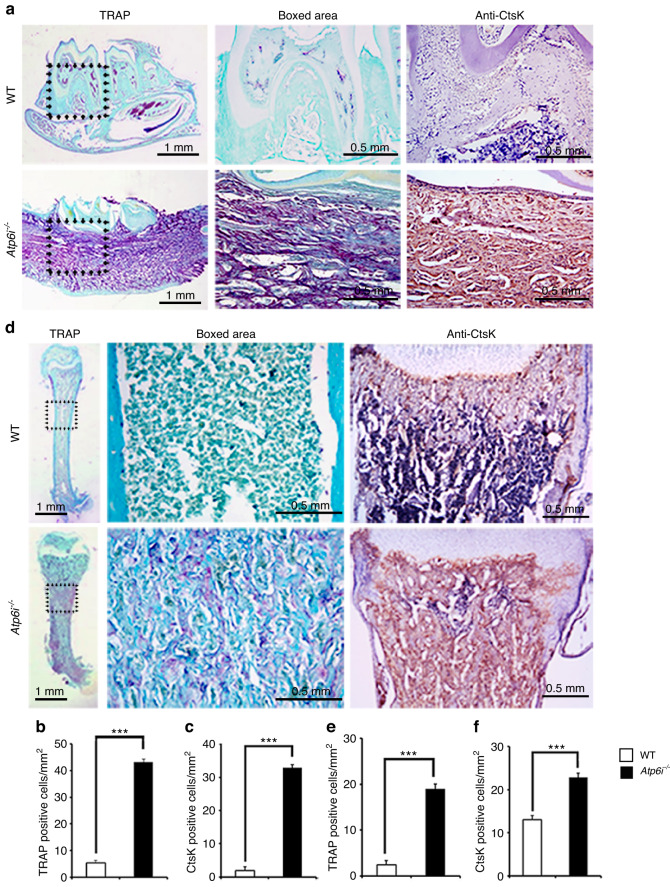
*Atp6i*^*−/−*^ mice exhibited arrested root formation, osteopetrosis phenotype due to impaired OC activity and increases the number of TRAP-positive osteoclasts in alveolar and femoral bone at 2 weeks. **a** TRAP and anti-CtsK IHC staining of representative mandibles in WT and *Atp6i*^*−/−*^ mice groups. The middle and right panels are the black dotted boxed area from the left panels. **b**, **c** Quantification data of TRAP and anti-CtsK IHC staining of WT and *Atp6i*^*−/−*^ mice groups. **d** TRAP and anti-CtsK IHC staining of representative femur in WT and *Atp6i*^*−/−*^ mice groups. The middle and the right panels are the black dotted boxed area from the left panels. **e**, **f** Quantification data of TRAP and anti-CtsK IHC staining of WT and *Atp6i*^−*/*−^ mice groups. *n* = 7 in each group; *** *P* < 0.001

**Fig. 2:**
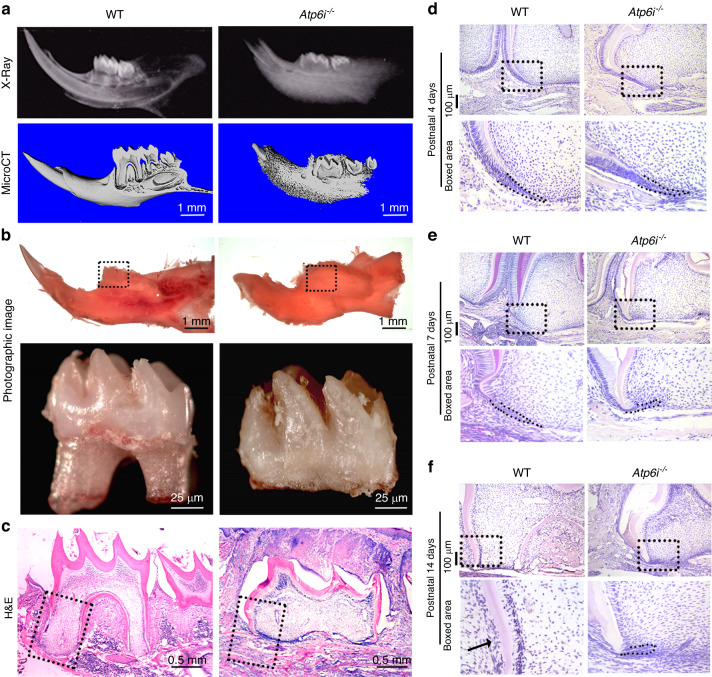
*Atp6i* knockout mice exhibit disrupted tooth root formation with truncated HERS progression. **a** Representative radiograph and micro-CT images show defective molar root formation and eruption in 3-week-old *Atp6i*^*−/−*^ mice compared to WT mice. **b** Representative photographic images show no tooth eruption (dotted box) and root development (first mandibular molar) in 3-week-old *Atp6i*^−*/*−^ mice compared to WT mice. **c** Representative H&E staining images of first mandibular molars showing normal tooth development in 3-week-old WT mice and impaired root formation in *Atp6i*^*−/−*^ mice of the same age. **d**–**f** Representative images of H&E staining of sagittal tooth sections from WT and *Atp6i*^*−/−*^ mice of 4 days (**d**), 7 days (**e**) and 14 days (**f**) PN. HERS is delineated by the dotted lines and arrow. *n* = 7 in each group

Using embryonic stem cells, we generated a targeted deletion of Atp6i at exons 2–5 for our Atp6i^−/−^ mouse model as described previously.^7^ Tartrate-resistant acid phosphatase (TRAP) staining and IHC staining for cathepsin K (CtsK) in the *Atp6i*^*−/−*^ mice revealed increased OC number in the alveolar bone surrounding the tooth (Fig. 1a–c). Consistently, the long bones of *Atp6i*^*−/*−^ mice had significantly increased TRAP-positive osteoclasts and Ctsk-positive staining compared to WT controls (Fig. 1d–f), which is due to the high density of the bone in *Atp6i*^*−/−*^ mice compared to WT. Radiographic and micro-computed tomography (µCT) analyses of the mandibles demonstrated arrested tooth root formation in the *Atp6i*^*−/−*^ mice as compared with littermate controls (Fig. 2a). Notably, photographic imaging and hematoxylin and eosin (H&E) staining confirmed absent molar root formation and blunted incisors in the *Atp6i*^*−/−*^ mice even at 3 weeks of age (Fig. 2b, c). The tooth crown formation was also mildly affected in the *Atp6i*^*−/−*^ mice, with a reduction of crown dentin thickness (Fig. 2a) and slight expansion of the crown predentin (Fig. 2c). Moreover, H&E staining of the first molars from WT and *Atp6i*^*–/–*^ mice at different ages (4–14 days post-natal) showed severe truncation of the HERS in the mutant teeth (Fig. 2d), which by comparison was elongated downward in WT mice at 7 days (Fig. 2e) which resulted in a full-length root by 14 days (Fig. 2f). We further characterized OC number and function in *Atp6i*^*−/*−^ mice. Human mutations in Atp6i lead to an OC-rich osteopetrosis phenotype in which there are abundant yet dysfunctional OCs.^30^

### Atp6i deficiency abolishes odontogenesis during tooth root development

**Fig. 3:**
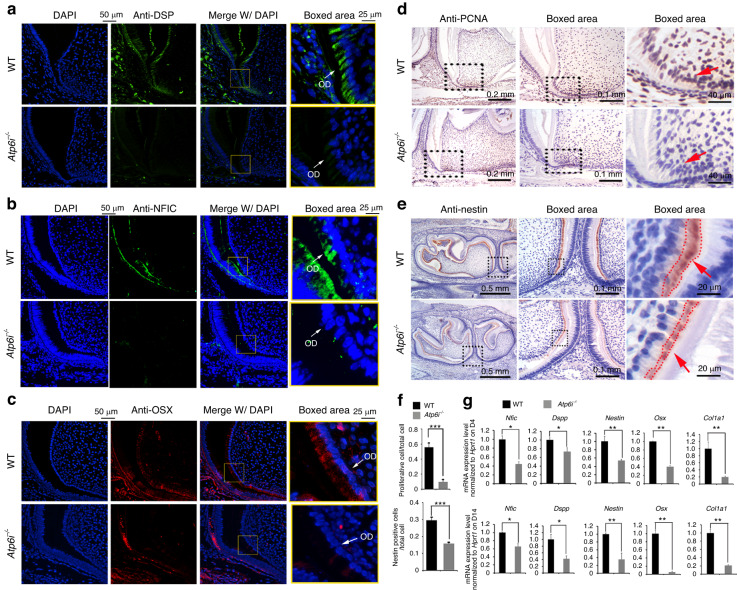
Atp6i deficiency abolishes odontogenic cell proliferation and odontoblast differentiation during tooth root development. **a**–**c** IF staining of DSP, NFIC, and OSX in mandibular first molars from 4 days PN WT and *Atp6i*^*−/−*^ mice. OD, Odontoblast (**d**, **e**) IHC staining of PCNA and nestin in WT and *Atp6i*^−*/*−^ mice at 4 days PN. **f** Quantification of PCNA- and nestin-positive cell ratios in WT and *Atp6i*^*−/*−^ mice groups. **g** qPCR analysis of the expression of odontoblast differentiation markers in alveolar bone-root samples from 4 and 14 day PN WT and *Atp6i*^*−/−*^ mice. *Hprt* was used as an endogenous control. *n* = 7 in each group; *P < 0.05; **P < 0.01; ***P < 0.001

Normal odontogenic cell proliferation and differentiation are essential for the development of the tooth root.^31^ Through proliferating cell nuclear antigen (PCNA) staining, we demonstrated that the proliferative activity of both cervical loop and ectomesenchymal cells of *Atp6i*^*−/−*^ mice were significantly decreased as compared with WT mice (Fig. 3d, f). Nestin, an intermediate filament family member, is involved in the generation of the unique odontoblast cellular processes.^22^ IHC staining showed a reduced expression level and region of nestin localization in the *Atp6i*^*−/−*^ mice (Fig. 3e, f). The expression of Nfic, Osx, and Dsp, which were clearly detectable in WT radicular odontoblasts, were only slightly detectable in *Atp6i*^*−/*−^ mice (Fig. 3a–c). Using tissue of the alveolar bone-root region, which included root, dental pulp, and alveolar bone, we confirmed that expression of *Nfic*, *Dspp*, *nestin*, *Osx*, and *Col1a1* were all significantly decreased in *Atp6i*^*−/*−^ mice at 4 and 14 days PN through qPCR analysis (Fig. 3g). Although the mixed tissue data reflected mRNA expression from the root and alveolar bone region, decreased levels of odontoblast-specific markers including *Nfic* and *Dspp* implied inhibited odontoblast differentiation in the mutant mice.

### Smad2/3 activation in Atp6i knockout mice is significantly suppressed

**Fig. 4:**
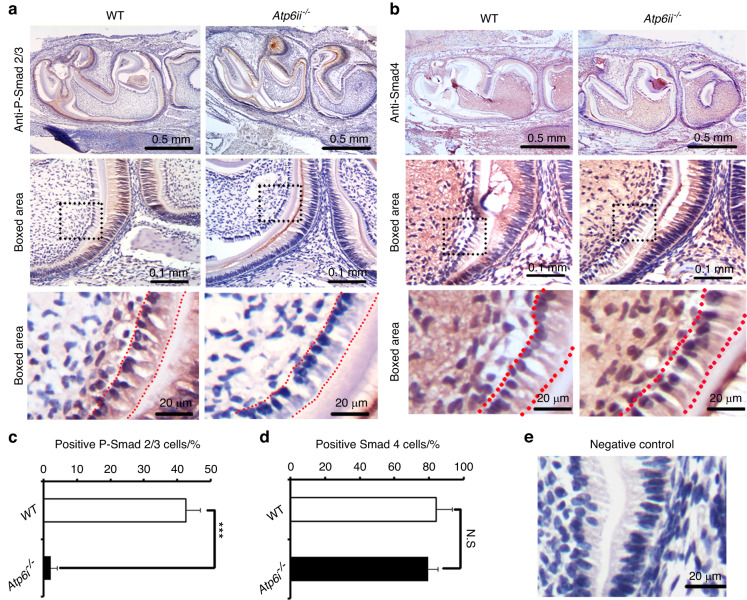
Smad2/3 activation in Atp6i Knockout mice is significantly suppressed. **a** IHC staining of p-Smad2/3 in odontoblasts of 4 day PN WT and *Atp6i*^*−/−*^ mice. **b** Quantification data of p-Smad2/3 positive odontoblasts in WT and *Atp6i*^*−/−*^ mice in (**a**). **c** IHC staining of Smad4 in odontoblasts in 4 days PN WT and *Atp6i*^*−/−*^ mice. **d** Quantification data of Smad4 positive odontoblasts in WT and *Atp6i*^*−/*−^ mice groups in (**c**). **e** Normal serum served as negative control. Dotted lines in (**a**, **c**) indicate the odontoblast cell layer. *n* = 7 in each group; **P < 0.01; ***P < 0.001

In order to establish the cause of the inhibited odontoblast differentiation, we examined important signaling pathways that regulate odontoblast differentiation in the *Atp6i*^*−/−*^ mice and found that activation of Smad2/3, regarded as an indicator of TGF-β activity, was severely attenuated in the polarized odontoblast layer of the *Atp6i*^*−/−*^ mice (Fig. 4a, b). In contrast, Smad4, a central intracellular effector of TGF-β signaling, showed no significant difference between the control and *Atp6i*^*−/−*^ mice (Fig. 4c, d). This data indicated that Atp6i deficiency may influence Smad2/3 nuclear translocation in TGF-β signaling.

### Atp6i deficient osteoclastic bone resorption-conditioned medium inhibits odontoblast differentiation and TGF-β1 signaling transduction in vitro

**Fig. 5:**
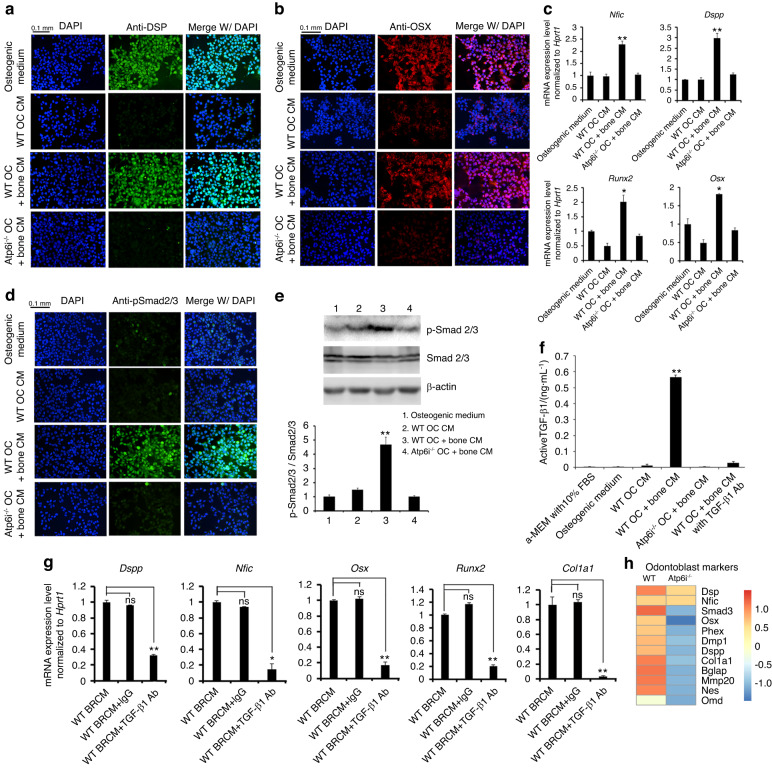
Atp6i deficient osteoclastic bone resorption-conditioned medium inhibits odontoblast differentiation and TGF-β1/ Smad2/3 signaling transduction in vitro. **a**, **b** Immunofluorescent staining of DSP and OSX in A4 pulp precursor cell line induced by different cell culture media for 3 days. **c** qPCR analysis of gene expression levels of odontoblast differentiation markers in odontoblast-like cells induced by different cell culture media for 7 days. *Hprt* was used as an endogenous control. **d** Immunofluorescent staining of pSmad2/3 in odontoblast-like cells induced by different cell culture media for 3 days. **e** Western blot of p-Smad2/3 expression in odontoblast-like cells induced by different cell culture media for 7 days. **f** Active TGF-β1 level in different cell culture media detected by Elisa; WT OC + bone CM versus other groups. **g** qPCR analysis of odontoblast differentiation markers gene expression levels in odontoblast-like cells induced by WT OC BRCM with addition of TGF-β1 neutralization antibody for 7 days. WT OC BRCM added with IgG was used as the control group. WT OC CM, WT osteoclast culture medium; WT OC + bone CM (WT BRCM), conditioned medium from WT osteoclast cultured with bone; *Atp6i*^−/−^ OC + bone CM, conditioned medium from *Atp6i*^−/−^ osteoclast cultured with bone; WT OC + bone CM with TGF-β1 Ab, WT OC BRCM added with TGF-β1 neutralization antibody. *n* = 7 in each group; *P < 0.05; **P < 0.01; ns, not significant. **h** Heatmap showing expression levels of odontoblast markers in P20 WT and Atp6i^−/−^ mice mandibles

As aberrant TGF-β signaling was detected in Atp6i^−/−^ odontoblasts, we further explored the mechanism underlying how OC dysfunction in *Atp6i*^−/−^ mice might be associated with the TGF-β signaling disturbance. Given that TGF-β1 was identified as a potent coupling growth factor, we hypothesized that this process of osteoclast-osteoblast coupling may be applied to osteoclast-odontoblast interactions. Thus, we conducted in vitro odontoblast differentiation assays using Bone Resorption-Conditioned Medium (BRCM). We first confirmed that WT splenocytes plated on mice calvarias could differentiate into mature and functional OCs that generated abundant erosion pits on bone slice surfaces (Supplementary Fig. 1B). In contrast, although *Atp6i*^−/−^ splenocytes could be induced into TRAP-positive OC on calvaria, these OCs were unable to resorb bone (Supplementary Fig. 1C). Next, we detected that BRCM from WT OCs cultured on bone slices led to significantly higher expression of DSP and OSX in in vitro-cultured A4 pulp precursor cells,^32^ while BRCM from either WT OCs cultured without bone slices or *Atp6i*^−/−^ OCs cultured on bone slices induced much lower DSP and OSX expression levels (Fig. 5a, b). qPCR analysis of odontoblast differentiation marker gene expression profiles (*Runx2, Nfic, Osx, Dspp*, and *Col1a1*) in the pulp precursor cell line treated with different types of cell culture media for 7 days confirmed that WT OC BRCM promoted the highest expression levels compared with osteogenic medium, OC-only conditioned medium, and *Atp6i*^−/−^ OC BRCM (Fig. 5c). These results indicated that functional OCs cultured on bone slices could promote odontoblast differentiation by releasing potential factor(s) from the bone matrix into the conditioned medium.

To explore whether TGF-β1 is potentially the released factor mediating OC-odontoblast communication, we detected p-Smad2/3 in the pulp precursor cell line cultured with different media including WT and *Atp6i*^−/−^ OC BRCM. Consistent with results observed in vivo, WT OC BRCM induced a higher level of p-Smad2/3 in odontoblast-like cells but a very low level in the *Atp6i*^−/−^ OC BRCM induced-group, as determined by IF staining (Fig. 5d) and Western blot (Fig. 5e). Moreover, ELISA results demonstrated clearly that a high level of active TGF-β1 was detected in WT OC BRCM, while *Atp6i*^−/−^ OC BRCM contained barely detectable levels of active TGF-β1 (Fig. 5f). To verify that TGF-β1 functions as the main factor in WT OC BRCM to induce odontoblast differentiation, we depleted TGF-β1 in WT OC BRCM by adding a neutralizing TGF-β1 specific antibody and found that the expression levels of odontoblast differentiation markers (*Col1a1, Dspp, Runx2, Nfic, and Osx*) were significantly inhibited in odontoblast-like cells (Fig. 5g). Consistently, RNA-seq analysis of mandibles including tooth, periodontium, and alveolar bone from P20 WT and Atp6i^−/−^ mice showed decreased expression levels of odontoblast markers such as Dmp1, Dspp, Bglap, Nes, and Omd, as well as decreased expression levels of Smad3 (Fig. 5h). Depletion of TGF-β1 in WT BRCM by antibody neutralization and immunoprecipitation was confirmed by ELISA assay (Fig. 5f). These results further demonstrate that the TGF-β1 /Smad2/3 signaling pathway plays a key role in regulating odontoblast differentiation and tooth root formation.

### Partial rescue of odontoblast differentiation in vitro and Atp6i^−/−^ tooth germ root formation in vivo with the addition of TGF-β1

**Fig. 6:**
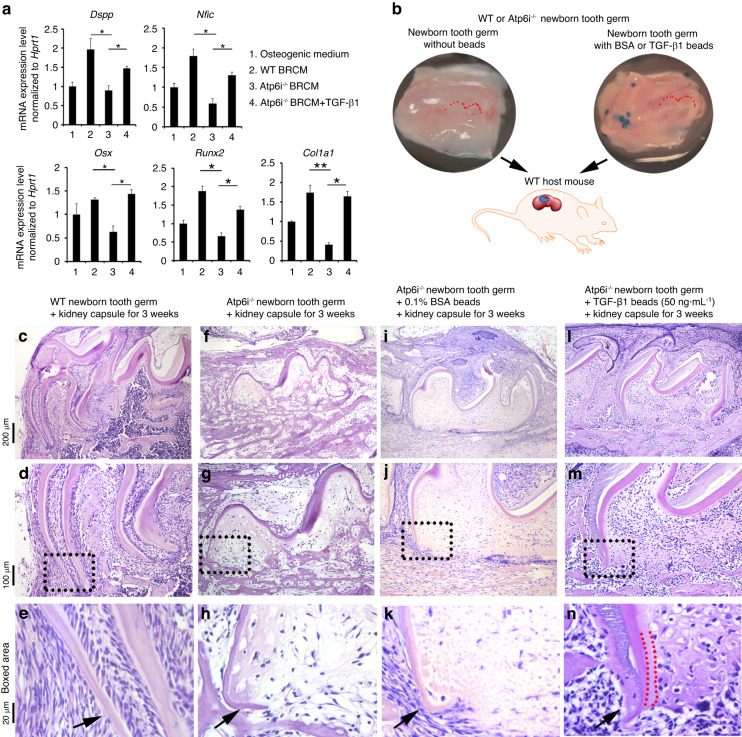
Rescue of odontoblast differentiation in vitro and *Atp6i*^*−/−*^ tooth germ root formation in vivo with addition of TGF-β1. **a** qPCR result of the expression of odontoblast differentiation markers in pulp precursor cell line induced by *Atp6i*^−/−^ OC BRCM with addition of TGF-β1 for 7 days. WT BRCM, conditioned medium from WT osteoclast cultured with bone; *Atp6i*^−/−^ BRCM, conditioned medium from *Atp6i*^*−/−*^ osteoclast cultured with bone. *Hprt* was used as an endogenous control. *n* ≥ 6 in each group; *P < 0.05; **P < 0.01. **b** Schematic diagram of the kidney capsule transplantation system. Newborn tooth germs alone or with beads were transplanted underneath WT host mouse kidney capsule for in vivo rescue experiment. Red-dotted lines outlined crown of the first molar in the tooth germ. **c**–**n** Representative images of H&E staining of newborn WT and *Atp6i*^*−/*−^ tooth germs cultured under kidney capsules. Newborn WT (**c**–**e**) and *Atp6i*^*−/−*^ (**f**–**h**) tooth germs were transplanted alone under kidney capsules, or with 0.1% BSA beads (**i**–**k**) or TGF-β1 beads (**l**–**n**) for 3 weeks. Arrows indicated root formation in each sample. Red-dotted sections in (**n**) outlined radicular dentin. *n* = 7 tooth germs per each group

As functional OCs can release TGF-β1 from the bone matrix and thus induce odontoblast differentiation in vitro, we next used TGF-β1 to rescue impaired odontoblast differentiation in vitro and *Atp6i*^−/−^ mice root formation in vivo. Firstly, we found the addition of active TGF-β1 into *Atp6i*^−/−^ OC BRCM significantly promoted the expression of odontoblast differentiation markers (*Osx, Runx2, Dspp, Col1a1*, and *Nfic*) compared to the low expression levels found in *Atp6i*^−/−^ OC BRCM-treated odontoblast-like cells (Fig. 6a). Therefore, the addition of TGF-β1 can rescue impaired odontoblast differentiation with BRCM of *Atp6i*^−/−^ OC in vitro. To test whether TGF-β1 can rescue root formation in the mutant mice, we transplanted tooth germs dissected from newborn WT and *Atp6i*^−/−^ mice under kidney capsules of WT host mice for 3 weeks with or without TGF-β1 beads (Fig. 6b). We found that *Atp6i*^−/−^ newborn tooth germs grafted alone to the kidney capsule showed no evidence of root development (Fig. 6f–h) in contrast to WT newborn tooth germs which almost fully developed roots (Fig. 6c–e), indicating that cytokines present in the bloodstream provided by WT host mice were not adequate to rescue the defected root formation within mutant mice. However, we detected root elongation in *Atp6i*^−/−^ samples transplanted with TGF-β1 beads (Fig. 6l–n) whereas there was no sign of root structure in *Atp6i*^−/−^ samples transplanted with BSA control beads (Fig. 6i–k). Although the odontoblasts in the root were not well organized, dentin was deposited in the root area of the *Atp6i*^−/−^ teeth (Fig. 6n). Notably, alveolar bone formation underneath the tooth germ also largely increased in TGF-β1-treated *Atp6i*^−/−^ samples (Fig. 6l) compared to the control groups (Fig. 6f, i), confirming the coupling effect of TGF-β1 in promoting bone formation in bone remodeling.^28^

## Discussion

In this study, we are the first to reveal that TGF-β1 released by osteoclastic bone resorption induces tooth root formation. We utilized Atp6i deficient mice as an osteopetrosis disease model to study tooth root formation and found that Atp6i knockout mice exhibit disrupted tooth root formation with truncated HERS progression as well as marked decreases in odontoblast markers Dspp, Nfic, Nestin, Osx, and Col1a1. Smad2/3 activation was significantly attenuated indicating suppressed TGF-β signaling in Atp6i^−/−^ odontoblasts, while Smad4 expression was not significantly different. RNA-seq analysis of mandibles from P20 WT and Atp6i^−/−^ mice showed decreased expression levels of odontoblast markers such as Dmp1, Dspp, Bglap, Nes, and Omd, as well as decreased Smad3 expression. Conditioned medium containing TGF-β1 promoted odontoblast differentiation, which was significantly blocked by anti-TGF-β1 antibody neutralization, whereas odontoblast differentiation and Smad2/3 activation were significantly attenuated by *Atp6i-*deficient OC conditioned medium. Importantly, ectopic TGF-β1 partially rescued root development and root dentin deposition of *Atp6i*^−/−^ tooth germs transplanted under the mouse kidney capsule. Collectively, our novel data is the first to demonstrate TGF-β1/Smad2/3 as a key signaling pathway regulating odontoblast differentiation and tooth root formation.

**Fig. 7:**
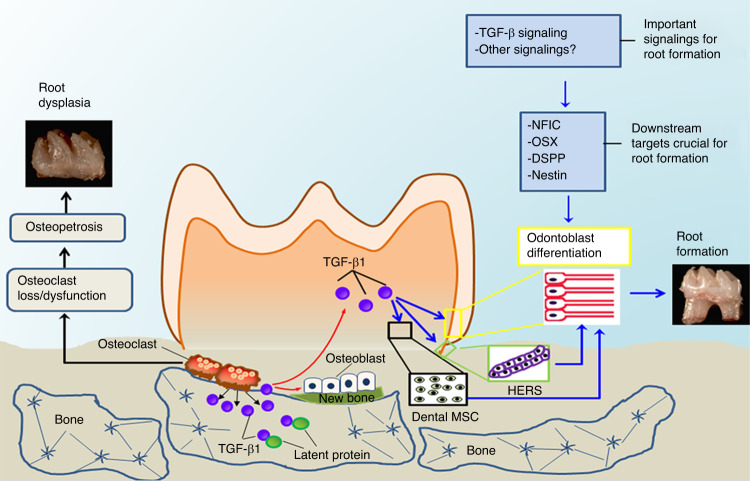
TGF-β1 as a key regulator of odontoblast differentiation and tooth root formation. Schematic diagram of the working hypothesis: TGF-β1 is released from bone matrix in response to osteoclastic bone resorption, and acts as a coupling growth factor in inducing radicular odontoblast differentiation and tooth root formation. TGF-β signaling plays an important role in regulating radicular odontoblast differentiation through affecting the expression of downstream targets (Nfic, Osx, Dspp and Nestin) in the process of root development. Particularly, TGF-β1 not only mediates OC-osteoblast communication and promotes bone formation, but also functions as an OC-odontoblast coupling factor which plays direct regulatory roles on odontoblasts, HERS, and may also on dental MSCs and eventually induces odontoblast differentiation and root formation. On the other hand, osteoclast loss/dysfunction often leads to osteopetrosis-associated root dysplasia resulted from impaired TGF-β1-mediated OC-odontoblast coupling in tooth root development. HERS, Hertwig’s epithelial root sheath

Tooth crown development has been studied extensively during past decades,^33–35^ yet tooth root development is not well understood. With the discovery of Nfic, a key regulator of radicular odontoblasts differentiation associated with root formation and independent of crown formation,^36^ it has become clear that the regulatory mechanisms underlying post-natal root formation are independent of crown formation. The discovery of Osx as a key downstream target of Nfic that also plays a critical role in root formation, but not crown formation, further demonstrates that tooth root and crown formation regulation is unique.^37^ Thus, we cannot apply the knowledge obtained from tooth crown studies when exploring the pathogenetic mechanism of root dysplasia in human osteopetrosis. Although the complexity of odontoblast differentiation and functional status at different anatomical areas within the pulp is not well understood, there is a growing evidence that the alveolar bone microenvironment has stronger regulatory effects on root development than it does on crown development.^16,37^ However, it is still unclear how local bone environments modify the expression of specific root regulatory genes, such as *Nfic* and *Osx*, and hence mediate radicular odontoblast differentiation. In the present study, we sought to decipher these underlying mechanisms by using the *Atp6i*^*−/−*^ osteopetrosis mouse model. Our data showed that dysfunctional OCs in the alveolar bone cannot release TGF-β1 from the bone matrix, leading to attenuated TGF-β and Smad2/3 signaling in radicular odontoblasts. As downstream targets of TGF-β signaling, expression of key odontoblast differentiation genes was inhibited, which resulted in impaired odontoblast differentiation and root formation in osteopetrosis (Fig. 7). We speculate that when the microenvironment in alveolar bone surrounding the tooth changes due to impaired osteoclast function, root formation of the tooth would be more dramatically affected as compared to that of crown formation.

Multiple lines of evidence suggest that TGF-β receptor signaling through activation of Smad2/3 is essential for odontoblast differentiation and root development.^22,31^ This is underscored by a study indicating that the loss of TGF-β signaling in odontoblast and bone-producing mesenchyme in *Osx-cre;Tgfbr2*^*fl/fl*^ mutant mice affected both odontoblast function and root formation.^25^ Moreover, it has been reported that exogenous TGF-β1 can induce odontoblast differentiation and dentin formation in dental papilla cells in vitro, which is consistent with our in vitro and in vivo rescue experiments. However, it is important to point out that TGF-β1 induces odontoblast differentiation mainly in the early differentiation stages, and that constant overexpression of TGF-β1 through the odontoblast mineralization stage results in the downregulation of Dspp which is crucial for dentin mineralization.^38,39^ This may account partly for the partial rescue of root formation by TGF-β1, suggesting an essential but not sufficient role of TGF-β1 to rescue root formation. Other molecular mechanisms including Nifc, Wnt, and parathyroid hormone-related peptide (PTHrP) are involved in modulating late odontoblast differentiation and mineralization in root development.^19,36,40^ We recently revealed that bone resorption deficiency affects tooth root development in RANKL mutant mice due to attenuated insulin-like growth factor-1 (IGF-1) signaling in radicular odontoblasts,^41^ which could be another factor that plays a key role in root formation besides TGF-β1. In addition, it is worth mentioning that the tooth germs harvested included the surrounding alveolar bone tissues that contain TGF-β1. However, release of TGF-β1 from the bone matrix relies on osteoclast-mediated bone resorption processes. As the function of the Atp6i^−/−^ OCs was entirely blocked, TGF-β1 was not able to be released from the bone matrix even with the presence of the alveolar bone.

The function of OCs in the developing tooth has been recognized primarily as opening a pathway through the jaw bone for tooth eruption.^42^ Previous reports have also suggested that tooth defects in OC loss/dysfunction mouse models are caused by a physical disturbance of osteopetrotic bone rather than the tooth itself.^43,44^ However, root development and tooth eruption are two coupled yet chronologically different processes that may involve independent regulatory mechanisms.^45^ Our discovery of disrupted odontoblast differentiation and signaling transduction as early as PN4 in the studied OC dysfunction mouse model implies a more complicated regulatory role for OCs related to the process of root formation. In fact, it is found that the typical treatment for autosomal recessive osteopetrosis, which is hematopoietic stem cell transplantation, can improve tooth eruption, yet issues with root formation still remain.^30^ Thus, we speculate that OCs play unique roles in tooth root formation and tooth eruption. This is supported by the finding that there is a major burst of osteoclastogenesis at PN3 and a minor burst at PN10 in the first mandibular molar, which coincide with the initiation of root formation (PN4) and tooth eruption (PN14). Based on our current study, we suggest that, besides providing an eruption pathway through alveolar bone by bone resorption, OCs function to release essential cytokines that regulate the differentiation and maturation of odontoblasts during tooth root formation. We speculate that even though hematopoietic stem cell transplantations were conducted in patients at as early as 6 weeks of age, it took a certain amount of time for the reconstruction of normal bone structure and recovery of functional OCs, which are critical for the initial stage of root formation. Thus, defective differentiation of radicular odontoblasts could not be reversed at the time of root formation, resulting in lack of root development in the patients after treatment.

## Conclusion

In summary, we used Atp6i deficient mice as an osteopetrosis disease model to study tooth root formation and found that loss of osteoclast function significantly impaired the proliferation and differentiation of radicular odontogenic cells responsible for root formation. In addition, we found that Smad2/3 activation was significantly attenuated, indicating that TGF-β signaling was suppressed in Atp6i-deficient odontoblasts. Conditioned medium containing TGF-β1 from WT osteoclastic bone resorption promoted odontoblast differentiation, which was significantly blocked by anti-TGF-β1 antibody neutralization, whereas odontoblast differentiation and Smad2/3 activation were significantly attenuated by *Atp6i*^−/−^ OC conditioned medium, indicating impaired Smad-dependent signaling. Importantly, ectopic TGF-β1 partially rescued root development and root dentin deposition of *Atp6i*^−/−^ tooth germs transplanted under the mouse kidney capsule. Collectively, our novel results are the first to demonstrate TGF-β1 /Smad2/3 as a key signaling pathway regulating odontoblast differentiation and tooth root formation through the process of OC-mediated bone resorption is critical for the normal development of the tooth root. This study provides an improved understanding of the regulatory mechanism of root formation leading to a more comprehensive knowledge of tooth morphogenesis and may lay the foundation for future tooth root regeneration studies.

## Materials and methods

### Animals

Atp6i^−/−^ mice with a C57BL/6J genetic background were previously generated by our lab.^7^ Male wild-type (WT) C57BL/6J mice (from Jackson Laboratory) were used as the control group. All animal experimentation was carried out according to the legal requirements of the Association for Assessment and Accreditation of the Laboratory Animal Care International and the University of Alabama at Birmingham Institutional Animal Care and Use Committee (IACUC) and Tulane University IACUC and followed all the recommendations found in ARRIVE (Animal Research: Reporting in Vivo Experiments) guidelines. Mice were bred in-house and euthanatized by CO_2_ asphyxiation. All mice were maintained under a 12 h light–dark cycle with ad libitum access to regular food and water.

### Harvest and preparation of samples

Animals were sacrificed by CO_2_ inhalation and harvested. The mandibles were removed and hemisected. After the removal of soft tissue, the left side of the jaw samples was fixed in 4% formaldehyde for 24 h and then stored in 70% ethanol before X-ray and Micro-CT analysis. The right side of the jaw samples was fixed in 4% paraformaldehyde, then de-calcified and prepared for paraffin embedding. The specimens were then serially sectioned and mounted according to standard procedures for histological analysis.

### Histological analysis

Hematoxylin & eosin (H&E) staining was performed as described.^46^ Tartrate-resistant acid phosphatase (TRAP) stain was used as a marker for osteoclasts using a commercial kit (Sigma).^47^

### Acridine orange staining

Acid production of osteoclast was determined using acridine orange staining following the method described previously.^47,48^

### Scanning electron microscopy analysis

Bone resorption capacity of osteoclast from WT and *Atp6i*^*−/−*^ mice was assessed as described.^9,49^

### Immunohistochemistry (IHC) and immunofluorescence (IF) analysis

Mandibular tooth root sections and in vitro-cultured A4 pulp precursor cells^32^ were examined by IHC and IF staining as described previously.^50^

### Preparation of bone resorption-conditioned medium (BRCM)

We isolated OC precursors from the spleen of newborn WT and *Atp6i*^−/−^ mouse as described previously.^46^ Mouse calvarias were harvested from 4 to 6-week-old mice and snap-frozen with liquid nitrogen to preserve bone matrix cytokines while eliminating live cells. Subsequently, WT or Atp6i^−/−^ splenocytes (1 × 10^5^ cells per well) were plated on bone slices seeded in 48-well tissue culture plates and cultured in α-modified MEM (GIBCO-BRL) supplemented with 10% (vol/vol) fetal bovine serum (FBS) (GIBCO-BRL) in the presence of 20 ng·mL^–1^ macrophage colony-stimulating factor (M-CSF; R&D Systems) for 24 h. Cells were then submitted to osteoclastogenesis from combined stimulation with 10 ng·mL^–1^ receptor activator of NF-κB ligand (RANKL; R&D Systems) and 10 ng·mL^–1^ M-CSF. The conditioned media from OC-mediated bone resorption were harvested at days 8 to 10 post-stimulation.

### RNA extraction and quantitative real-time PCR (RT-qPCR)

For tooth root sample RNA extraction, the prepared samples were first transferred to the tube prefilled with beads (Nextadvance Company, USA) and homogenized using a Bullet blender (Nextadvance Company, USA). The RNA extraction from root samples or odontoblasts was performed using TRIzol reagent (Invitrogen, USA) with the standard procedure. The extracted RNA was used for reverse transcription using a RevertAid Reverse Transcriptase kit (Thermo Scientific, Waltham, MA). Real-time quantitative PCR was performed as described previously^48^ using primers purchased from Invitrogen as listed (see Appendix Table 1). Briefly, cDNA fragments were amplified with Sybr green fast advanced master mix (Applied Biosystems, Foster City, CA) and detected by a Step-One real-time PCR system (Applied Biosystems). The mRNA expression level of the *Hprt1* housekeeping gene was used as an endogenous control and specific mRNA expression levels were calculated as a ratio to *Hprt1* level. RNA extraction for odontoblast cells was performed directly using TRIzol reagent and followed the above procedures.

### RNA samples preparation and RNA-seq

RNA-sequencing and analysis was performed as previously described.^51^ In brief, total mRNA was isolated using TRIzol reagent (Invitrogen Corp., Carlsbad, CA) from mice mandibles following the manufacturer’s protocol and was submitted to Admera Health (South Plainsfield, NJ) who assessed sample quality with the Agilent Bioanalyzer and prepared the library using the NEBnext Ultra RNA–Poly-A kit. Libraries were analyzed using Illumina next generation sequencing and relative quantification was provided by Admera Health. Read counts were subjected to paired differential expression analysis using the R package DESeq2.^52^

### Western blotting analysis

Mandibular root samples from 4-day and 14-day WT and *Atp6i*^−/−^ mice were dissected and rinsed in chilled PBS, and immediately frozen at −80 °C until used for protein extraction. For preparing the samples for western blot analysis, we harvested the root samples cutting from the alveolar bone-root region of the WT mice mandible, which included root, dental pulp and alveolar bone surrounding the root and exclude crown. As there is no tooth root for *Atp6i*^−/−^ mice, we harvested the root samples from the same area as from the WT mice in order to compare the protein expression level of the same alveolar bone-root area. Protein extraction from root samples or odontoblasts and western blotting were performed as previously described^50,53^ and a Fluor-S Multi-Imager with Multi-Analyst software (Bio-Rad) was used for visualization and quantification. The rabbit anti-Atp6i antibody was previously generated by our lab^7^ and was used at a 1:1 000 dilution. phospho-Smad2/3 protein and Smad2/3 protein levels were analyzed with the following primary antibodies: rabbit-anti-phospho-Smad2/3 (1:1 000; Cell signaling) and rabbit-anti-Smad2/3 (1:1 000; Cell signaling). Horseradish peroxidase-linked anti-rabbit IgG (7074 S, Cell signaling) was used to visualize the reaction.

### Enzyme-linked immunosorbent assay (ELISA)

ELISA was used to determine active TGF-β1 level in different cell culture media and conditioned media with the Human/Mouse TGF beta 1 ELISA kit (eBioscience, USA) according to the manufacturer’s instructions. Results were expressed as ng cytokine/ml.

### Kidney capsule transplantation

Kidney capsule transplantation was carried out as described.^21,54^ For the rescue experiment, TGF-β1 or BSA beads were placed adjacent to the tooth grafts under the kidney capsule. Bead preparation was carried out as described previously.^16,55^

### Statistical analysis and data quantification

Experimental data are reported as mean ± standard deviation (SD). Results were analyzed with the two-tailed Student’s *t* test or ANOVA analysis. Mann–Whitney *U* test was used for the non-parametric test. *P* values < 0.05 or *U* values > 1.96 were considered significant. Data quantification analyses were performed by using the NIH ImageJ Program as described.^49^

### Supplementary information

Supplemental Materials

## Data Availability

The RNA-Seq data are available upon request. Contact: Yi-Ping Li, Department of Pathology and Laboratory Medicine, Tulane University School of Medicine, E-mail: yli81@tulane.edu. All other data are contained within the manuscript.
