# Supplementary material for: Atp6i deficient mouse model uncovers transforming growth factor-β1 /Smad2/3 as a key signaling pathway regulating odontoblast differentiation and tooth root formation
Source: Int J Oral Sci. 2023 Aug 21;15:35. doi: 10.1038/s41368-023-00235-2 (PMC10440342; doi:10.1038/s41368-023-00235-2)
Supplement: Supplementary file 1 — Supplemental Materials [file 41368_2023_235_MOESM1_ESM.docx]

**Atp6i deficient mouse model uncovers TGF-β1 /Smad2/3 as a key signaling pathway regulating odontoblast differentiation and tooth root formation**

Jue Wang^1, 2^, Abigail McVicar^3^, Yilin Chen^3^, Hong-Wen Deng^4^, Zhihe Zhao^2^, Wei Chen^1,3, #^, Yi-Ping Li^1,3, #^

**Supplemental Materials and Methods**

**Animals**

*Atp6i^-/-^* mice with a C57BL/6J genetic background were previously generated by our lab ^1^. Male wild-type (WT) C57BL/6J mice (from Jackson Laboratory) were used as the control group. All animal experimentation was carried out according to the legal requirements of the Association for Assessment and Accreditation of the Laboratory Animal Care International and the University of Alabama at Birmingham Institutional Animal Care and Use Committee (IACUC) and Tulane University IACUC, and followed all recommendations of ARRIVE (Animal Research: Reporting in Vivo Experiments) guidelines. Mice were bred in-house and euthanatized by CO2 asphyxiation. All mice were maintained under a 12 h light–dark cycle with ad libitum access to regular food and water.

**Harvest and Preparation of Samples**

Animals were sacrificed by CO_2_ inhalation and harvested. The mandibles were removed and hemisected. After removal of soft tissue, the left side of jaw samples were fixed in 4% formaldehyde for 24 hours then stored in 70% ethanol prior to X-ray and Micro-CT analysis. The right side of jaw samples were fixed in 4% paraformaldehyde, then were de-calcified and prepared for paraffin embedding. The specimens were then serially sectioned and mounted according to standard procedures for histological analysis.

**Radiographic Procedures**

X-ray analysis and microcomputed tomography (MicroCT) radiography were performed as previously described ^2^ at the University of Alabama at Birmingham Small Animal Bone Phenotyping Core associated with the Center for Metabolic Bone Disease.

**Histological Analysis**

Hematoxylin & eosin (H&E) staining was performed as described ^3^. Tartrate-resistant acid phosphatase (TRAP) stain was used as a marker for osteoclasts using a commercial kit (Sigma) ^4^. Multinucleated TRAP-positive cells appear as dark purple cells and were counted by light microscopy.

**Acridine Orange Staining**

Acid production of osteoclast was determined using acridine orange staining following the method described previously ^4,5^. Splenocytes were harvested from WT and *Atp6i^-/-^* mice of 2-week old and cultured in a-MEM containing 10% FBS with 10 ng/mL recombinant macrophage colony-stimulating factor (M-CSF) and 10 ng/mL recombinant receptor activator of nuclear factor κB ligand (RANKL). After 5 days’ stimulation, cells were incubated in α-MEM containing 5 ug/ml of acridine orange (Sigma) for 19 minutes at 37°C, washed three times with PBS solution, and chased for 10 minutes in fresh media with no acridine orange. Cell images were obtained with a fluorescence microscope with a 490nm excitation filter and a 525nm arrest filter.

**Scanning Electron Microscopy Analysis**

Bone resorption capacity of osteoclast from WT and *Atp6i^-/-^* mice was assessed as described ^2,6^. 5 X 10^4^ cells/well of splenocytes were seeded on bovine cortical bone slices in 24-well plates and stimulated for 5 days with M-CSF and RANKL. The bone slices were then harvested and cells adhering to the bone slices were subsequently removed with 0.25 M ammonium hydroxide and mechanical agitation. Bone slices were visualized by scanning electron microscopy (SEM) using a Philips 515 SEM (Department of Materials Science and Engineering, UAB). The data were quantified using ImageJ analysis software by measuring the percentage of the areas resorbed in three random resorption sites.

**Immunohistochemistry (IHC) and Immunofluorescence (IF) Analysis**

Mandibular tooth root sections and *in vitro*-cultured A4 pulp precursor cells ^7^ were examined by IHC and IF staining as described previously ^8^. The following antibodies were used: mouse monoclonal anti-cathepsin K (CtsK, 1:200; Santa Cruz, USA), goat polyclonal anti-proliferating cell nuclear antigen (PCNA, 1:400; Santa Cruz, USA), chicken polyclonal anti-nestin (1:10000; Aves LABS, USA), rabbit polyclonal anti-dentin sialoprotein (DSP) (1:200; Santa Cruz, USA), mouse monoclonal anti-nuclear factor I C (NFIC) (1:200; Abcam, USA), goat polyclonal anti-OSX (1:200; Santa Cruz, USA), rabbit monoclonal anti-phospho-Smad2/3 (1:200; Cell Signaling, USA), and mouse monoclonal anti-Smad4 (1:200; Santa Cruz, USA).

**Preparation of Bone Resorption-Conditioned Medium (BRCM)**

We isolated OC precursors from spleen of newborn WT and *Atp6i^-/-^* mouse as described previously ^3^. Mouse calvarias were harvested from 4 to 6 week-old mice and snap-frozen with liquid nitrogen to preserve bone matrix cytokines while eliminating live cells. Subsequently, WT or *Atp6i^-/-^* splenocytes (1x10^5^ cells per well) were plated on bone slices seeded in 48-well tissue culture plates and cultured in α-modified MEM (GIBCO-BRL) supplemented with 10% (vol/vol) fetal bovine serum (FBS) (GIBCO-BRL) in the presence of 20 ng/mL macrophage colony-stimulating factor (M-CSF; R&D Systems) for 24 hours. Cells were then submitted to osteoclastogenesis from combined stimulation with 10 ng/mL receptor activator of NF-κB ligand (RANKL; R&D Systems) and 10 ng/mL M-CSF. The conditioned media from OC-mediated bone resorption were harvested at days 8 to 10 post stimulation.

***In Vitro* Differentiation Assays of the Pulp Precursor Cell Line**

A4 pulp precursor cells were plated into 12-well plates at a density of 4X10^5^ cells per well and cultured in following media: osteogenic medium, Dulbecco’s modified Eagle’s medium (DMEM) supplemented with 10% FBS, 2 mmol/L L‑glutamine, 100 units/ml penicillin/streptomycin, 50 μg/ml ascorbic acid, and 10 mM sodium β-glycerophosphate (Sigma); WT OC conditioned medium: 20% conditioned media from WT OC culturing on plate (without bone slices) diluted with DMEM; WT OC + bone conditioned medium (or WT BRCM): 20% conditioned media from WT OC culturing on bone slice diluted with DMEM; *Atp6i^-/-^*  OC + bone conditioned medium (or *Atp6i^-/-^* BRCM): 20% conditioned media from *Atp6i^-/-^* OC culturing on bone slice diluted with DMEM. Three repeated wells per group were set and experiments were performed in triplicate. Culture media were changed every 2 days and cells were harvested for IF and qPCR analysis at corresponding time points.

Antibody neutralization and immunoprecipitation were conducted to deplete TGF-β1 from BRCM. 2 ug/ml TGF-β1 monoclonal antibody (BD Biosciences, #555052, USA) was added to the conditioned medium (BRCM), incubated overnight in 4°C, and then adsorbed to Protein G Magnetic Beads (NEB). BRCM added with mouse IgG (Jackson Immunology) was used as a control.

For *in vitro* rescuing assay, recombinant mouse TGF-β1 (5 ng/mL; Cell Signaling, #5231, USA) was added to *Atp6i^-/-^* BRCM (*Atp6i^-/-^* BRCM + TGF-β1) to treat plated pulp precursor cell line. Before use, the TGF-β1 was treated with 20 mM citrate, pH 3.0 to make a stock solution and activate the protein according to the manufacturer's instructions.

**Kidney Capsule Transplantation**

Kidney capsule transplantation was carried out as described previously ^9,10^. Tooth germs were harvested from newborn WT and *Atp6i^-/-^* mice and transplanted under the kidney capsule according to standard procedures. Then 3 weeks after transplantation, the WT host mice were sacrificed, and the explants were processed for histologic analysis. For rescue experiment, TGF-β1 or BSA beads were placed adjacent to the tooth grafts under the kidney capsule. Bead preparation was carried out as described ^11,12^. Affi-Gel blue agarose beads (Bio-Rad, USA) were washed in PBS and then incubated in recombinant mouse TGF-β1 (50 ng/mL; Cell Signaling, USA) or 0.1% bovine serum albumin (BSA) overnight at 4°C prior to use.

**RNA Extraction and Quantitative Real-Time PCR (RT-qPCR)**

For tooth root sample RNA extraction, the prepared samples were first transferred to the tube prefilled with beads (Nextadvance Company, USA) and homogenized using a Bullet blender (Nextadvance Company, USA). The RNA extraction from root samples or odontoblasts was performed using TRIzol reagent (Invitrogen, USA) with the standard procedure. The extracted RNA was used for reverse transcription using a RevertAid Reverse Transcriptase kit (Thermo Scientific, Waltham, MA). Real-time quantitative PCR was performed as described previously ^5^ using primers purchased from Invitrogen as listed (see Appendix Table 1). Briefly, cDNA fragments were amplified with Sybr green fast advanced master mix (Applied Biosystems, Foster City, CA) and detected by a Step-One real-time PCR system (Applied Biosystems). The mRNA expression level of the *Hprt1* housekeeping gene was used as an endogenous control and specific mRNA expression levels were calculated as a ratio to *Hprt1* level. RNA extraction for odontoblast cells was performed directly using TRIzol reagent and followed the above procedures.

**RNA samples preparation and RNA-seq.** RNA-sequencing and analysis was performed as previously described ^13^. In brief, total mRNA was isolated using TRIzol reagent (Invitrogen Corp., Carlsbad, CA) from mice mandibles following the manufacturer's protocol and was submitted to Admera Health (South Plainsfield, NJ) who assessed sample quality with the Agilent Bioanalyzer and prepared the library using the NEBnext Ultra RNA - Poly-A kit. Libraries were analyzed using Illumina next generation sequencing and relative quantification was provided by Admera Health. Read counts were subjected to paired differential expression analysis using the R package DESeq2 ^14^.

**Western Blotting Analysis**

Mandibular root samples from 4-day and 14-day WT and *Atp6i^-/-^* mice were dissected and rinsed in chilled PBS, and immediately frozen at -80°C until used for protein extraction. For preparing the samples for western blot analysis, we harvested the root samples cutting from the alveolar bone-root region of the WT mice mandible, which included root, dental pulp and alveolar bone surrounding the root and exclude crown. As there is no tooth root for *Atp6i^-/-^* mice, we harvested the root samples from the same area as from the WT mice in order to compare the protein expression level of the same alveolar bone-root area. Protein extraction from root samples or odontoblasts and western blotting were performed as previously described ^8,15^ and a Fluor-S Multi-Imager with Multi-Analyst software (Bio-Rad) was used for visualization and quantification. The rabbit anti-Atp6i antibody was previously generated by our lab ^1^ and was used at a 1:1000 dilution. phospho-Smad2/3 protein and Smad2/3 protein levels were analyzed with the following primary antibodies: rabbit-anti-phospho-Smad2/3 (1:1000; Cell signaling) and rabbit-anti-Smad2/3 (1:1000; Cell signaling). Horseradish peroxidase-linked anti-rabbit IgG (7074S, Cell signaling) was used to visualize the reaction.

**Enzyme-linked Immunosorbent Assay (ELISA)**

ELISA was used to determine active TGF-β1 level in different cell culture media and conditioned media with the Human/Mouse TGF beta 1 ELISA kit (eBioscience, USA) according to the manufacturer’s instructions. Results were expressed as ng cytokine/ml.

**Statistical Analysis and Data Quantification**

Experimental data are reported as mean ± standard deviation (SD). Results were analyzed with the two-tailed Student’s t-test or ANOVA analysis. Mann-Whitney U test was used for the non-parametric test. P values <0.05 or U values >1.96 were considered significant. Data quantification analyses were performed by using the NIH ImageJ Program as described ^2^.

**Supplemental Table**

**Supplemental Table 1. Primers used for qRT-PCR**

| Primers used for qRT-PCR | | |
| --- | --- | --- |
| Gene | Forward Primers (5’-3’) | Reverse Primers (5’-3’) |
| *Atp6i* | CACAGGGTCTGCTTACAACTG | CGTCTACCACGAAGCGTCTC |
| *Nfic* | GGAACCGGACCCAACTTCTC | CGTCCTCTTCCATCGAGCC |
| *Dspp* | AGTTCGATGACGAGTCC | GTCTTCTCCCGCATGT |
| *Runx2* | AGTGCTCTAACCACAGTCCATGCA | TACAAACCATACCCAAGTACCTGTTT |
| *Nestin* | CACACCTCAAGATGTCCC | GAAAGCCAAGAGAAGCCT |
| *Osx* | CTGGGGAAAGGAGGCACAAAGAAG | GGGTTAAGGGGAGCAAAGTCAGAT |
| *Col1a1* | CTTGGTGGTTTTGTATTCGATGAC | GCGAAGGCAACAGTCGCT |
| *Hprt1* | GGTGGAGATGATCTCTCAACTTTAA | GGGAAAGCAAAGTTTGCATTGTT |

**Supplemental Figure**


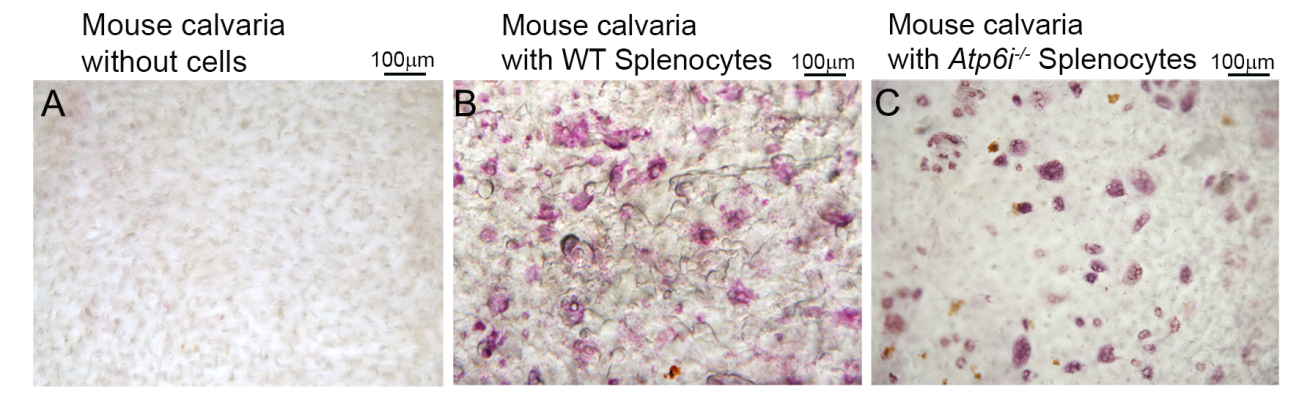


**Supplemental Figure 1. Atp6i deficiency impaired bone resorption function of osteoclast cultured on calvaria.** Representative images of TRAP staining of mouse calvaria cultured with WT osteoclasts (**B**) or *Atp6i^-/-^* osteoclasts (**C**) derived from mouse splenocytes by M-CSF and RANKL induction. Mouse calvaria without cells (**A**) served as the control. n = 7 in each group.

**References**

1 Li, Y. P., Chen, W., Liang, Y., Li, E. & Stashenko, P. Atp6i-deficient mice exhibit severe osteopetrosis due to loss of osteoclast-mediated extracellular acidification. *Nat. Genet.* **23**, 447-451, doi:10.1038/70563 (1999).

2 Gao, B. *et al.* Inhibiting periapical lesions through AAV-RNAi silencing of cathepsin K. *J. Dent. Res.* **92**, 180-186, doi:10.1177/0022034512468757 (2013).

3 Chen, W. *et al.* C/EBPα regulates osteoclast lineage commitment. *Proceedings of the National Academy of Sciences* **110**, 7294-7299, doi:10.1073/pnas.1211383110 (2013).

4 Chen, W. *et al.* Novel pycnodysostosis mouse model uncovers cathepsin K function as a potential regulator of osteoclast apoptosis and senescence. *Human molecular genetics* **16**, 410-423 (2007).

5 Yang, S. & Li, Y.-P. RGS10-null mutation impairs osteoclast differentiation resulting from the loss of [Ca2+] i oscillation regulation. *Genes & development* **21**, 1803-1816 (2007).

6 Ma, J. *et al.* RNA interference-mediated silencing of Atp6i prevents both periapical bone erosion and inflammation in the mouse model of endodontic disease. *Infect Immun* **81**, 1021-1030, doi:10.1128/iai.00756-12 (2013).

7 Lacerda-Pinheiro, S. *et al.* Concomitant multipotent and unipotent dental pulp progenitors and their respective contribution to mineralised tissue formation. *Eur Cell Mater* **23**, 371-386 (2012).

8 Hao, L. *et al.* A small molecule, Odanacatib, inhibits inflammation and bone loss caused by endodontic disease. *Infect. Immun.*, doi:10.1128/iai.01713-14 (2015).

9 Huang, X., Xu, X., Bringas, P., Hung, Y. P. & Chai, Y. Smad4‐Shh‐Nfic signaling cascade–mediated epithelial‐mesenchymal interaction is crucial in regulating tooth root development. *J. Bone Miner. Res.* **25**, 1167-1178 (2010).

10 Pavía-Jiménez, A., Tcheuyap, V. T. & Brugarolas, J. Establishing a human renal cell carcinoma tumorgraft platform for preclinical drug testing. *Nature protocols* **9**, 1848-1859 (2014).

11 Chai, Y. *et al.* A mouse mandibular culture model permits the study of neural crest cell migration and tooth development. *International Journal of Developmental Biology* **42**, 87-94 (2003).

12 Hosoya, A., Kim, J.-Y., Cho, S.-W. & Jung, H.-S. BMP4 signaling regulates formation of Hertwig’s epithelial root sheath during tooth root development. *Cell Tissue Res.* **333**, 503-509 (2008).

13 Tang, C. Y. *et al.* Runx1 is a central regulator of osteogenesis for bone homeostasis by orchestrating BMP and WNT signaling pathways. *PLoS Genet* **17**, e1009233, doi:10.1371/journal.pgen.1009233 (2021).

14 Love, M. I., Huber, W. & Anders, S. Moderated estimation of fold change and dispersion for RNA-seq data with DESeq2. *Genome Biol* **15**, 550, doi:10.1186/s13059-014-0550-8 (2014).

15 Chen, W. *et al.* Cbfbeta deletion in mice recapitulates cleidocranial dysplasia and reveals multiple functions of Cbfbeta required for skeletal development. *Proc. Natl. Acad. Sci. U. S. A.* **111**, 8482-8487, doi:10.1073/pnas.1310617111 (2014).
